# Risk factors associated with preterm birth among mothers delivered at Lira Regional Referral Hospital

**DOI:** 10.1186/s12884-023-06120-4

**Published:** 2023-11-23

**Authors:** Tom Etil, Bosco Opio, Bernard Odur, Charles Lwanga, Leonard Atuhaire

**Affiliations:** 1 School of Statistics and Planning, Makerere University, Kampala, Uganda; 2 Faculty of Public Health, Lira University, Lira, Uganda

**Keywords:** Factors associated, Preterm birth, Lira Regional Referral Hospital

## Abstract

**Background:**

The World Health Organization (WHO) defines Preterm Birth (PTB) as “a live birth taking place before the expected 37 weeks of gestation”. Annually, approximately 15 million infants are born prematurely, constituting significantly to infant mortality during the initial four weeks of life, responsible for 40% of deaths among children under the age of five. Evidently, preterm deliveries have contributed to 46% of admissions to the neonatal intensive care unit (NICU) at Lira Regional Referral Hospital (LRRH) over the past three years. Paradoxically, while the prevalence of preterm births remains high, there is a lack of documented information regarding the underlying risk factors. Consequently, the primary objective of this study was to assess the potential risk factors associated with preterm birth at LRRH.

**Methods:**

An analytical cross-sectional research was undertaken at LRRH, employing a quantitative methodology. The study utilized secondary data obtained from a total of 590 comprehensive maternal medical records, of deliveries that occurred at the facility between April 2020 and July 2021. The collected data underwent analysis using STATA version 17 software. To identify predictors of preterm birth, a Logistic regression model was applied, yielding adjusted odds ratios (AOR) alongside 95% confidence intervals (CI). The significance level was set at *p* < 0.05 to establish statistical significance. Furthermore, assessments for multicollinearity and model fitness were conducted using the Variance Inflation Factor (VIF) and linktest, respectively.

**Results:**

The prevalence of preterm delivery among mothers who gave birth at LRRH stood at 35.8%. The outcomes of logistic regression analysis revealed that maternal employment status had a statistically significant association with preterm birth (AOR = 0.657, *p* = 0.037, 95%CI: 0.443–0.975); having a baby with low birth weight (AOR = 0.228, *p* < 0.001, 95% CI: 0.099–0.527) and experiencing preeclampsia (AOR = 0.142, *p* < 0.001, 95% CI: 0.088–0.229) were also identified as significant predictors of preterm birth in the study.

**Conclusions and recommendations:**

The occurrence of preterm delivery is significantly higher (35.8%) among mothers who gave birth at LRRH when compared to the national average (13.6%). The prevalence of preterm birth among mothers was linked to factors such as employment status, delivery of low birth weight infants, and the presence of preeclampsia. Consequently, the research proposes a set of recommendations. Firstly, the Ministry of Health (MoH) should evaluate the present state of readiness within the healthcare system to effectively handle cases of preterm birth both within medical facilities and the community. Secondly, the Ministry of Gender, Labour, and Social Development should leverage Labor Officers to implement and uphold the regulations stipulated in the Employment Act and Labor Laws.

**Supplementary Information:**

The online version contains supplementary material available at 10.1186/s12884-023-06120-4.

## Introduction

Preterm birth (PTB), as defined by the World Health Organization (WHO), refers to “a live birth taking place prior to the completion of 37 weeks of gestation” [1]. Annually, approximately 15 million infants are delivered prematurely, and this global pattern is experiencing an upward trajectory [1]. Notably, PTB emerged as the primary cause of neonatal mortality within the initial four weeks of life, ranking as the second leading factor behind under-five deaths on a global scale; prematurity is responsible for 40% of under-five fatalities [2]. Geographically, preterm birth prevails across 184 nations, displaying incidence rates ranging from 5 to 18% among all newborns. Specifically, Africa and South Asia jointly contribute to 60 to 85% of all instances of preterm births [3]. Comparing demographics, approximately 12% of newborns in less affluent nations are born prematurely, in contrast to 9% in more economically prosperous countries [1].

Preterm birth rates in Sub-Saharan Africa have been investigated, revealing estimates of 10.9% in Gambia and 12% in Tanzania [4]. Within global rankings, Uganda holds the 28th position for preterm births, standing at 13.6 per 1000 live births. Tragically, 25% of the 27 deaths per 1000 live births of newborns can be directly attributed to preterm births. In light of the new global infant mortality goals aiming for fewer than 10 deaths per 1000 live births by 2035, Uganda must intensify its efforts to reduce preterm birth rates [4]. Achieving this objective would demand a targeted intervention on addressing preventable causes of preterm birth in low-income nations [4]. While factors such as demographics, social circumstances, obstetric care, and pregnancy-related conditions have been implicated in preterm births worldwide, it’s important to note that these risks differ according to geographical regions, leading to marked disparities between affluent and low-income countries [1, 4, 5].

To address the issue of premature births, the Ministry of Health, operating under the Government’s initiative, has established a nationwide framework for pregnancy care and assistance. This includes bolstering emergency medical services and referral systems, enhancing maternal, neonatal, and child health provisions across all tiers of healthcare, expanding access to sexual and reproductive health amenities with a particular emphasis on family planning and age-appropriate information, and elevating the quality of national laboratory and diagnostic services [6]. Additionally, the United States Agency for International Development (USAID) through its Regional Health Integration to Enhance Services-North, Lango Activity (RHITES-N, Lango), provides support to hospitals like the LRRH in terms of maternal healthcare services. Despite the implementation of these measures, data shows that over the past three years, 46% of admissions to the Neonatal Intensive Care Unit (NICU) at LRRH were attributed to preterm deliveries [7]. While, prior researches focusing on various contexts have produced inconclusive findings regarding the predictors of preterm birth, there is a notable absence of documented evidence concerning predictors of premature birth specifically within Lira district. Given this research gap, the current study aims to evaluate the risk factors linked to preterm birth at LRRH, with the ultimate goal of pinpointing potential interventions capable of mitigating the occurrence of preterm births.

## Methodology

### Data source and study design

The collection of secondary data involved the retrieval of information from the medical documents (specifically, prenatal cards and registers) of mothers who gave birth at LRRH during the period from April 2020 to July 2021. A cross-sectional sample of 590 comprehensive records of mothers who gave birth at LRRH was examined.

### Study variables and measurement

The dependent variable in this study was preterm birth, dichotomized as birth occurring before 37 weeks (preterm birth) and at 37 weeks or later (term birth). Accordingly, a mother who gave birth before 37 weeks was classified as having experienced preterm birth and assigned a code of 1, while a mother who delivered at 37 weeks or beyond was categorized as having undergone term birth and assigned a code of 0.

The independent variables included various aspects of family socio-demographics. Age was measured in completed years and subsequently grouped into three categories during the analysis: <18 years, 18–30 years, and 31 years or older. Marital status was stratified into four categories: married, single, divorced, and widow/widower. Education level was divided into four categories: no education, primary education, secondary education, and tertiary education. Employment status was dichotomized as employed or not employed. Location was classified as rural or urban. Other independent variables included Body Mass Index (BMI) measured in kg/m², which was segmented into four categories during analysis: underweight, normal weight, overweight, and obese. Substance use was categorized as active drug user or not an active drug user. Maternal factors were also considered, with mode of delivery categorized as either normal delivery or caesarean section. Antenatal Care (ANC) attendance was categorized as either less than 3 times or 3 times or more. Anemia was divided into two categories: <10 g/dl and ≥ 10 g/dl. History of abortion was classified as either having experienced abortion or never having experienced abortion. The birth weight of the mother’s baby was grouped into “yes” (< 2,500 g) or “no” (≥ 2,500 g). Comorbidities were classified as, whether present or absent. HIV status was categorized as sero-positive or sero-negative. Parity was stratified as having fewer than 4 children or having 4 or more children. Inter-pregnancy interval was segmented as less than 24 months or 24 months or more. Previous preterm birth was dichotomized as “yes” or “no,“ and preeclampsia was similarly classified as present or absent. Furthermore, fetal factors were considered, including the sex of the child, categorized as male, female, or both (in the case of twins of different sexes). Pregnancy outcome was classified as singleton or twins. Congenital abnormalities were recorded as “yes” or “no.“

### Data analysis

The data analysis was conducted using STATA version 17.0 software. In the initial univariate analysis, frequencies and percentages were computed to describe the variables considered in the study. At bivariate analysis, associations were examined using the Pearson Chi-square test with a significance level set at *p* < 0.05. Factors that demonstrated significance in this analysis were selected for inclusion in the subsequent multivariate analysis. The purpose of the multivariate analysis was to estimate the individual net effects of each independent factor on the dependent variable. Notably, due to the low prevalence of preterm births (ranging from 5 to 18%) as indicated in the literature, which categorizes it as a rare event, the binary complementary log-log model was a potential choice for isolating net effects, as opposed to the logistic and probit models. However, an evaluation of three Link functions (Logistic, Complementary log-log, and Probit) was performed to determine their suitability for fitting the data. Based on the Akaike’s Information Criteria (AIC) and Bayesian Information Criteria (BIC), the logistic model exhibited the lowest AIC and BIC values, rendering it the most plausible model for identifying predictors of preterm birth. Subsequently, two diagnostic tests were executed: a multicollinearity test and a model fitness test employing the Variance Inflation Factor (VIF) and link test, respectively.

### Ethics and consent to participate

This study employed the Research Ethics Committee of Makerere University’s School of Statistics and Planning to obtain approval and oversee the research process, ensuring adherence to both the regulations set forth by the Uganda National Council for Science and Technology (UNCST) and other international guidelines concerning the involvement of human subjects. The study made use of aggregated data sourced from a hospital, for which institutional authorization had been obtained to carry out the research. Informed consent was not pursued, as the data extracted did not contain any elements that could potentially reveal the identities of the patients. Physical copies of the data were securely stored and accessible solely to the research team. Similarly, electronic databases were safeguarded with passwords, with access restricted exclusively to the research team. Lastly, a request for a waiver of consent was submitted to Lira Regional Referral Hospital, an entity established to ensure the protection of study participants.

### Limitations

The research employed pre-existing data gathered at the establishment. The occurrence of the Covid-19 pandemic influenced the quantity of mothers who gave birth at the facility during the specified time frame (April 2020 to May 2021). To tackle this issue, the investigation period was prolonged until July 2021. Certain instances of insufficient or absent information, notably within the records, were faced. Nevertheless, this challenge was managed by incorporating information from antenatal cards to supplement the absent data.

## Results

### Background characteristics and differentials in preterm births by Socio-demographic, maternal and fetal characteristics of the mothers

Table 1 shows the distribution of participants based on socio-demographic, maternal, and fetal attributes. Some entries exhibited a lack of approximately 10% information in specific variables, causing a deviation from the total count of 590 respondents. These incompletely recorded instances were omitted from the analysis. The tabulated data indicates that out of the total 590 female participants, roughly 36% experienced preterm deliveries. The largest segment (82%) of the respondents fell within the age range of 18 to 30, approximately 56% were not engaged in employment, and nearly 48% resided in rural areas. In relation to marital status, a significant majority of the mothers (87%) were married, while an estimated 6% possessed limited or no literacy. Concerning maternal characteristics, nearly 9% of the mothers attended less than three antenatal care sessions, and around 40% underwent Caesarean section deliveries. Moreover, about 14% of the mothers tested positive for HIV, 6% gave birth to low weight infants, and almost 23% experienced preeclampsia. In regard to fetal attributes, a considerable proportion of the mothers (97%) had single babies, 54% of whom were female, and roughly 3% exhibited congenital abnormalities.

**Table 1:** Differentials in preterm births by Socio-demographic, maternal and fetal characteristics of the mothers (N = 590)

Variable		Frequency	%	No. of preterm birth	%	${\varvec{\chi }}^{2}$ (p-value)
*Preterm birth status*	Term birth ($\ge$37 weeks )	379	64.2			
	Preterm birth (< 37weeks)	211	35.8			
*Socio-demographic characteristics*						
Age group	< 18	17	2.9	8	47.1	2.62 (0.269)
	18–30	479	82.2	164	34.2	
	31 and above	87	14.9	36	41.4	
BMI	Underweight	6	6	3	50	4.84 (0.184)
	Normal	248	248	81	32.7	
	Overweight	254	254	99	39	
	Obese	19	19	10	52.6	
Marital status	Married	513	87.2	180	35.1	0.83 (0.842)
	Single	14	2.4	5	35.7	
	Divorced	54	9.2	22	40.7	
	Widow/Widower	7	1.2	2	28.6	
Education Level	No Education	32	5.5	10	31.3	4.0 (0.262)
	Primary	203	34.6	66	32.5	
	Secondary	302	51.6	120	39.7	
	Tertiary	50	8.5	15	30	
Location	Rural	267	47.7	86	32.2	1.75 (0.186)
	Urban	293	52.3	110	37.5	
Employment^1^	Employed	257	43.7	105	40.9	4.9 (0.027*)
	Not employed	331	56.3	106	32	
Drug use (Smoking/Alcohol)	Not active	379	64.3	137	36.2	0.05 (0.825)
	Active	210	35.7	74	35.2	
*Maternal Characteristics*						
ANC Attendance	< 3 times	55	9.3	21	38.2	0.15 (0.702)
	$\ge$3 times	534	90.7	190	35.6	
Mode of delivery	Normal delivery	346	60.1	114	33	3.76 (0.053)
	Caesarian section	230	39.9	94	40.9	
Parity	< 4 children	539	92.4	185	34.3	4.37 (0.037*)
	$\ge$4 children	44	7.6	22	50	
History of abortion	Aborted	60	10.2	27	45	2.45 (0.118)
	Never aborted	529	89.8	184	34.7	
Inter-pregnancy interval (in months)	< 24 months	66	11.2	27	40.9	0.86 (0.355)
	$\ge$24 months	524	88.8	184	35.1	
Low birth weight baby	Yes (< 2500g)	37	6.3	26	70.3	20.46 (< 0.001*)
	No ($\ge$2500g)	553	93.7	185	33.5	
HIV status	Sero-positive	80	13.9	32	40	0.69 (0.407)
	Sero-negative	497	86.1	175	35.2	
Comorbidity	Present	41	7.1	10	24.4	2.62 (0.105)
	Absent	538	92.9	199	37	
Anemia/Level of hemoglobin	< 10g/dl	13	2.3	4	30.8	0.17 (0.678)
	$\ge$10g/dl	561	97.7	204	36.4	
Preeclampsia	Yes	134	22.8	92	68.7	81.34 (< 0.001*)
	No	455	77.2	119	26.2	
Previous preterm	Yes	63	10.8	33	52.4	8.66 (0.003*)
	No	521	89.2	175	33.6	
*Fetal Characteristics*						
Sex of child	Male	259	43.9	94	36.3	6.98 (0.031*)
	Female	320	54.2	109	34.1	
	Both Male and Female^2^	11	1.9	8	72.7	
Pregnancy outcome	Singleton	570	96.6	197	34.6	10.56 (< 0.001*)
	Twins	20	3.4	14	70	
Congenital abnormalities	Yes	19	3.4	8	42.1	0.35 (0.557)
	No	546	96.6	194	35.5	

**NB**: *All variables do not sum to 590 due to missing data;* * Significance at 5%; X^2^**=** Chi-square

^1^ Formal employment (salary and wage earners)

^2^ In case of twins of different sexes

Table 1 also provides an overview of variations in preterm birth across different socio-demographic, maternal, and fetal characteristics. The table demonstrates that several factors are significantly linked to preterm birth, including maternal employment, parity, low birth weight babies, preeclampsia, previous preterm births, the sex of the child, and pregnancy outcome. Differences in preterm birth based on socio-demographic characteristics reveal that maternal employment (chi-square = 4.90, *p* = 0.027) is notably associated with preterm birth.

An analysis of maternal characteristics demonstrates significant associations with preterm birth. These include parity (chi-square = 4.37, *p* = 0.037), low birth weight baby (chi-square = 20.46, *p* < 0.001), preeclampsia (chi-square = 81.34, *p* < 0.001), and previous preterm births (chi-square = 8.66, *p* = 0.003).

Furthermore, an analysis of fetal characteristics indicates that the sex of the child (chi-square = 6.98, *p* = 0.031) and pregnancy outcome (chi-square = 10.56, *p* < 0.001) are also significantly associated with preterm birth.

### Multivariate analysis

The factors that were found to be statistically and significantly associated with preterm birth during the bivariate analysis were further examined in multivariate logistic regression. These factors were; employment, parity, low birth weight baby, preeclampsia, previous preterm births, pregnancy outcomes, and the sex of the child. In Table 2, it is evident that the factors indicating a significant association with preterm birth were: maternal employment, the presence of a low birth weight baby, and the occurrence of preeclampsia. The results from the logistic regression analysis indicated the predictive potential of certain socio-demographic factors in relation to preterm birth. Specifically, the analysis indicated that maternal employment played a role in predicting preterm birth. Accordingly, mothers who were not employed exhibited a roughly 34% reduced odds of giving birth to a preterm baby when compared to those who were employed (AOR = 0.657, *p* = 0.037, 95% CI: 0.443–0.975).

**Table 2:** Multivariate Analysis of factors associated with preterm birth

Variable		Logistic regression Model		
		AOR	p–value	[95% CI]
*Socio–demographic Characteristics*				
Employment	Employed	1.000	.	.
	Not employed	0.657	0.037**	0.443–0.975
*Maternal Characteristics*				
Mode of delivery	Normal delivery	1.000	.	.
	Caesarian section	1.026	0.902	0.681–1.545
Parity	< 4 children	1.000	.	.
	$\ge$4 children	1.708	0.150	0.823–3.542
Low birth weight baby	Yes (< 2500g)	1.000	.	.
	No ($\ge$2500g)	0.228	< 0.001***	0.099–0.527
Preeclampsia	Yes	1.000	.	.
	No	0.142	< 0.001***	0.088–0.229
Previous preterm	Yes	1.000	.	.
	No	0.588	0.110	0.307–1.128
*Fetal Characteristics*				
Sex of child	Male	1.000	.	.
	Female	0.704	0.089*	0.470–1.055
	Both male and female	1.640	0.639	0.207–13.007
Pregnancy outcome	Single tone	1.000	.	.
	Twins	3.825	0.090*	0.810–18.060
Constant		21.488	< 0.001***	7.278–64.449

*** p < 0.01, ** p < 0.05, * p < 0.1; AOR = Adjusted odds ratio; CI = Confidence interval

Examining maternal factors, the results revealed significant associations between preterm birth and having a baby with low birth weight and experiencing preeclampsia. In this regard, mothers who gave birth to babies weighing 2500 g or more displayed a nearly 77% reduced odds of having a preterm birth, in comparison to those who gave birth to babies weighing less than 2500 g (AOR = 0.228, *p* < 0.001, 95% CI: 0.099–0.527). Furthermore, mothers without preeclampsia exhibited an approximately 86% reduced odds of experiencing preterm birth when contrasted with mothers who had preeclampsia (AOR = 0.142, *p* < 0.001, 95% CI: 0.088–0.229).

However, fetal factors such as the sex of the child, pregnancy outcome, and the presence of a congenitally abnormal baby were not found to have a statistically significant association with preterm birth.

## Discussions

### Prevalence of preterm birth

The prevalence of preterm birth among mothers who delivered in LRRH was 35.8%. This indicated high prevalence compared to 13.6% in Mulago National Referral Hospital, Uganda [4], 18.3% in Nairobi, Kenya [8], 17.5% in a rural district hospital, Rwanda [9], 24.4% in Kilimanjaro Christian Medical Centre, Northern Tanzania [10], 25.9% in Jimma University Specialized Teaching & Referral Hospital, South–West Ethiopia [11], 24.3% in Mansoura University Hospital, Egypt [12], and 12.3% in Fafen Zone, Somalia area, Eastern Ethiopia [13]. The high prevalence of preterm birth in LRRH could be due to poor quality of antenatal care. This is a result of inadequate equipment such as antenatal ultrasound machines to identify fetal antenatal conditions and skills to manage these diagnoses, inadequate skills for the early identification of mothers at risk of preterm birth for timely management, inadequate multiple micronutrient supplementation, poor referral systems, inadequate and poor health infrastructures, inadequate health supplies, and health system structural factors. These technical, interpersonal, resource, and infrastructural factors impede the provision and experience of good quality maternity care at health facilities [6].

### Socio–demographic factors

The study anticipated that socio-demographic characteristics, such as age, BMI, marital status, education level, location, employment, and drug use (smoking/alcohol), would not exhibit a significant association with preterm birth among mothers delivered at LRRH. However, the findings revealed that employment emerged as a noteworthy predictor of preterm birth. According to the results, mothers who were unemployed demonstrated a significant difference (AOR = 0.657, *p* = 0.037, 95% CI: 0.443–0.975), being 0.657 times less likely to deliver preterm babies compared to those who were employed. This outcome aligns with studies conducted in Indonesia, which identified that working mothers faced a 16.2 times higher risk of delivering late preterm infants (LPI) in comparison to housewives (OR = 16.2; 95% CI: 2.315-123.444) [14]; in Mulago Hospital, Uganda, being unemployed (AOR = 0.36, 95%CI: 0.15–0.86, *p* = 0.021) was associated with a 64% reduction in the likelihood of experiencing preterm birth [4]. Similarly, in Cyprus, long working hours (OR: 3.77, 95% CI: 2.08–6.84) were about 4 times linked to preterm birth [15]. At Mansoura University Hospital, Egypt, increased risks were also observed between long working hours and temporary contracts, and the risk of preterm delivery (AOR = 2.36, CI: 1.18–7.78) and (AOR = 1.98, CI: 1.72–8.74) respectively [12]. However, the findings of this study are not corroborated by research conducted in Nigeria, which revealed that maternal occupation did not significantly affect gestational age (χ²=10.143, *p* = 0.428) and birth weight (χ²=16.807, *p* = 0.079) at delivery. Nevertheless, it did significantly affect stillbirth rates (χ²=28.134, *p* = 0.002) [16]. Furthermore, the nature of employment might impact a pregnant woman differently, depending on whether it involves manual or labor-intensive work. Similarly, a heavy workload could subject an expectant mother to stress, potentially leading to pregnancy complications and resulting in preterm delivery.

### Maternal factors

The study hypothesized that antenatal care (ANC) visits, mode of delivery, parity, history of abortion, inter–pregnancy interval, mothers with low birth weight babies, HIV status, comorbidity, anemia, preeclampsia, and previous preterm births are not significantly associated with preterm birth. However, this study found that mothers with low birth weight babies and preeclampsia were statistically significant predictors of preterm birth. The statistical analysis indicated that a mother with a low birth weight baby was a statistically significant predictor of preterm birth (AOR = 0.228, *p* < 0.001, 95% CI: 0.099–0.527). Accordingly, mothers who gave birth to babies with a weight of ≥2500 g were 0.228 times less likely to experience preterm birth than those who gave birth to babies weighing < 2500 g. This finding aligns with studies conducted at Shire Suhul General Hospital in Northern Ethiopia. Those studies discovered that mothers with a history of bearing neonates weighing less than 2500 g, including the most recent birth (AOR: 2.78, 95% CI: 1.39–5.55), were 2.8 times more likely to have preterm deliveries compared to their counterparts [17]. Similarly, a study at Jimma University Specialized Teaching and Referral Hospital in South West Ethiopia demonstrated that a history of low birth weight (OR = 0.085, CI: 0.04–0.18, *p* < 0.001) increased the likelihood of preterm delivery by a factor of 0.085 compared to those without a history of poor birth outcomes [11]. Further studies conducted in Abu Dhabi, United Arab Emirates, revealed that low birth weight babies (AOR = 17.62, CI: 11.05–28.10, *p* < 0.001) were nearly 18 times more likely to experience preterm births than their counterparts [5]. Additionally, in Public Hospitals of Fafen Zone, Somali Region, Eastern Ethiopia, newborns with birth weights less than 2500 g (AOR = 3.78, 95% CI: 1.55–9.84, *p* < 0.001) had a 3.78 times higher likelihood of being delivered preterm than babies with birth weights of ≥2500 g [13]. These findings could be attributed to various factors, including intrauterine growth restriction resulting from genetic factors and uterine infections, inadequate prenatal nutrition, chronic health conditions like diabetes, heart problems, and high blood pressure, placental issues, or maternal infections preventing proper oxygen and nutrient delivery to the fetus. Such conditions may contribute to the occurrence of preterm births and low birth weight babies [13, 17].

Additionally, preeclampsia was a statistically significant predictor of preterm birth (AOR = 0.254, *p* < 0.001, 95% CI: 0.185–0.348). This implies that mothers without preeclampsia were 0.254 times less likely to experience preterm birth than those with preeclampsia. This result is consistent with studies conducted at Mukalla Maternity and Childhood (MCH) Hospital in Yemen, which revealed that mothers with pre-eclampsia (AOR = 4.120; CI: 1.818–9.340, *p* < 0.001) were 4.12 times more likely to deliver preterm babies than those without [18]. Similarly, at Mulago Hospital in Uganda, mothers with preeclampsia (AOR = 16.24, 95% CI: 3.11–84.70, *p* < 0.001) were 16 times more likely to have a preterm birth than mothers without preeclampsia [4]. In Nanjing Maternity and Child Health Care Hospital, China, the odds of preterm birth among mothers with preeclampsia were 2.46 times higher (AOR = 2.46, 95% CI: 1.78–3.40, *p* < 0.001) than for mothers without the condition [19]. Likewise, in a regional hospital in Accra, Ghana, mothers with pre-eclampsia/eclampsia (AOR = 3.4, 95% CI: 1.0-11.9, *p* < 0.001) were 3.4 times more likely to experience preterm delivery than those without preeclampsia [20]. Preeclampsia may result from pregnancy complications characterized by high blood pressure, poor nutrition, high body fat, insufficient blood flow to the uterus, genetic factors, and signs of damage to other organ systems, most frequently affecting the liver and kidneys.

### Fetal factors

The study also hypothesized that fetal factors (such as the sex of the child, pregnancy outcome, and congenital abnormalities status) are not associated with preterm birth. However, this study revealed that no fetal factors were associated with preterm birth, which contradicts studies conducted in Public Hospitals of the central zone, Tigray, Ethiopia. These previous studies showed that neonates with congenital/birth defects (AOR = 3.19, 95% CI: 1.22, 8.34, *p* < 0.05) were three times more likely than those without any birth defects to experience preterm birth [21]. Similarly, research conducted at Shire Suhul General Hospital in Northern Ethiopia found that visible physical neonatal congenital anomalies in the most recent baby (AOR = 10.4; 95% CI: 1.66–65.2, *p* < 0.05) increased the odds of preterm birth occurrence compared to normal babies [17]. This discrepancy could be attributed to the interaction of genetic and environmental risk factors contributing to preterm delivery in Ethiopia, which may not be a prevalent issue in Uganda, especially in Lira District [21].

## Conclusions

This study examined the occurrence of preterm birth (PTB) and its related determinants within the context of deliveries at LRRH. The prevalence of preterm birth among mothers who gave birth at LRRH was documented at 35.8%. Specifically, the likelihood of preterm delivery was found to be lower among unemployed mothers in comparison to their employed counterparts. Similarly, mothers whose babies had a birth weight of less than 2500 g were observed to have a higher probability of delivering preterm compared to those whose babies had a normal birth weight. Conversely, the presence of preeclampsia was associated with a greater probability of preterm birth, whereas mothers without preeclampsia exhibited a reduced likelihood of delivering preterm babies.

## Recommendations

Basing on the key findings from the study, the followings recommendations were made:

1. To address the elevated occurrence of preterm birth within the LRRH and Lango sub-region, it is imperative for the Ministry of Health to evaluate the existing state of the healthcare system’s readiness to handle preterm births. This involves devising comprehensive plans and making necessary preparations, particularly in terms of procuring essential equipment. Furthermore, conducting training and mentorship programs aimed at imparting vital managerial and clinical proficiencies is essential. These programs are designed to empower frontline healthcare providers in promptly identifying and effectively managing expecting mothers who face a heightened risk of preterm birth. A crucial aspect of this initiative involves bolstering the referral system, ensuring a seamless pathway for timely and appropriate care. Concurrently, elevating the quality of antenatal care necessitates the implementation of routine antenatal ultrasounds. This step aims to enhance the identification of fetal antenatal conditions, contributing to improved care. Additionally, providing multiple micronutrient supplementation to pregnant mothers at risk of preterm birth emerges as a fundamental strategy to augment the overall quality of antenatal care.
2. Expectant employed mothers within the area must receive safeguarding from the implementation of the Employment Act and Labor Laws by the Ministry of Gender, Labour, and Social Development. This can be achieved by deploying Labor Officers, a measure aimed at addressing the concern of premature childbirth among working mothers.
3. To effectively reduce the occurrence of preterm birth and its associated consequences among mothers with preeclampsia and low birth weight infants in the LRRH and the broader Lango sub-region, it is crucial for healthcare professionals to recognize expectant mothers who are susceptible to delivering prematurely. This should be followed by the implementation of high-quality medical interventions, community-based health education, as well as informative awareness initiatives.

## Suggestions for further research

The study emphasized the necessity for additional quantitative and qualitative investigation into the factors contributing to preterm birth. The comprehensive information gathered from maternal participants and other relevant sources will enable the identification of unexplored variables, thus influencing the development of treatments and policy strategies.

### Electronic supplementary material

Below is the link to the electronic supplementary material.

Supplementary Material 1

## Data Availability

Data is available with the corresponding order on request.
