# Correction: Risk factors associated with preterm birth among mothers delivered at Lira Regional Referral Hospital

**DOI:** 10.1186/s12884-023-06163-7

**Published:** 2023-12-11

**Authors:** Tom Etil, Bosco Opio, Bernard Odur, Charles Lwanga, Leonard Atuhaire

**Affiliations:** 1 School of Statistics and Planning, Makerere University, Kampala, Uganda; 2 Faculty of Public Health, Lira University, Lira, Uganda

**Correction: BMC Pregnancy Childbirth 23, 814 (2023)**

10.1186/s12884-023-06120-4

Following publication of the original article [1], the authors reported an error in affiliations for the authors, namely Bosco Opio and Charles Lwanga. The correct affiliation for Charles Lwanga is School of Statistics and Planning, Makerere University, Kampala while for Bosco Opio is Faculty of Public Health, Lira University, Lira, Uganda.

The author group has been updated above and the original article [1] has been corrected.
