# Supplementary material for: Risk factors associated with preterm birth among mothers delivered at Lira Regional Referral Hospital
Source: BMC Pregnancy Childbirth. 2023 Nov 23;23:814. doi: 10.1186/s12884-023-06120-4 (PMC10666300; doi:10.1186/s12884-023-06120-4)
Supplement: Supplementary file 1 — Supplementary Material 1 [file 12884_2023_6120_MOESM1_ESM.docx]

**Appendices: Model Selection Criteria, Collinearity Statistics and Linktest results**

# **Appendix I: Goodness of Fit/Model Selection Criteria**

| **Model** | **AIC** | **BIC** |
| --- | --- | --- |
| Logistic regression | 632.227 | 675.507 |
| Complimentary log log | 635.801 |  |
| Probit | 633.009 | 676.289 |

# **Appendix II: Collinearity Statistics**

| **Variable** | **VIF** | **SQRT VIF** | **Tolerance** | **R-Squared** |
| --- | --- | --- | --- | --- |
| Age group | 1.17 | 1.08 | 0.8562 | 0.1438 |
| BMI | 1.08 | 1.04 | 0.9283 | 0.0717 |
| Marital Status | 1.12 | 1.06 | 0.8957 | 0.1043 |
| Education Level | 1.54 | 1.24 | 0.6505 | 0.3495 |
| Location | 2.25 | 1.5 | 0.4452 | 0.5548 |
| Employment Status | 1.97 | 1.4 | 0.5076 | 0.4924 |
| Drug Use | 1.09 | 1.04 | 0.9183 | 0.0817 |
| ANC Attendance | 1.07 | 1.04 | 0.9309 | 0.0691 |
| Mode of delivery | 1.08 | 1.04 | 0.9284 | 0.0716 |
| Parity | 1.17 | 1.08 | 0.855 | 0.145 |
| History of abortion | 1.48 | 1.22 | 0.6746 | 0.3254 |
| Inter–pregnancy interval | 1.58 | 1.26 | 0.6343 | 0.3657 |
| History of giving low birth weight baby | 1.24 | 1.11 | 0.8056 | 0.1944 |
| HIV status | 1.04 | 1.02 | 0.9595 | 0.0405 |
| Comorbidity | 1.13 | 1.06 | 0.8877 | 0.1123 |
| Anaemia/Level of hemoglobin | 1.07 | 1.04 | 0.9319 | 0.0681 |
| Preeclampsia | 1.12 | 1.06 | 0.8941 | 0.1059 |
| Previous preterm | 1.22 | 1.11 | 0.8188 | 0.1812 |
| Sex of child | 1.15 | 1.07 | 0.8717 | 0.1283 |
| Pregnancy outcome | 1.08 | 1.04 | 0.9217 | 0.0783 |
| Congenitally defected baby | 1.13 | 1.07 | 0.8814 | 0.1186 |

**Mean VIF 1.27**

**Appendix III: The linktest**

| **Outcome** | | **Coef.** | **Std.Err.** |  | **z** | **P>z** | **[95%Conf** | **Interval]** |
| --- | --- | --- | --- | --- | --- | --- | --- | --- |
| _hat | | 0.976 | 0.103 |  | 9.510 | 0.000 | 0.775 | 1.177 |
| _hatsq | | -0.077 | 0.088 |  | -0.880 | 0.378 | -0.249 | 0.094 |
| _cons | | 0.084 | 0.153 |  | 0.550 | 0.581 | -0.215 | 0.383 |
|  |  | | | | | | | |
